# Delayed Serum Creatine Kinase Elevation After Snakebite: Serial Changes and Clinical Correlates of Myotoxicity in a Korean Tertiary-Care Cohort

**DOI:** 10.3390/jcm15156079

**Published:** 2026-08-05

**Authors:** Young Chan Park, Oh Hyun Kim, Kang Hyun Lee, Sun Ju Kim

**Affiliations:** Department of Emergency Medicine, Yonsei University Wonju College of Medicine, Wonju 26426, Republic of Korea; bgjs27@gmail.com (Y.C.P.); ohkim@yonsei.ac.kr (O.H.K.); ed119@yonsei.ac.kr (K.H.L.)

**Keywords:** snakebite, serum creatine kinase, myotoxicity, rhabdomyolysis, antivenom, local progression, serial monitoring

## Abstract

**Background/Objectives:** Initially normal serum creatine kinase (CK) activity may not exclude evolving muscle injury after a snakebite. We evaluated delayed CK elevation, clinical correlates, antivenom exposure, and hospital-day trajectories. **Methods:** We retrospectively analyzed 499 snakebite encounters at a tertiary hospital in South Korea. Delayed elevation was defined as serum CK ≥1000 U/L at the first or second follow-up after an initial value <1000 U/L. Incomplete follow-up was assessed using a lower bound, inverse-probability weighting (IPW), weight truncation, and outcome-model standardization. **Results:** Among 433 encounters with initially normal CK levels, 258 were retested, and 45 developed delayed elevation (17.4%; 95% confidence interval, 13.3–22.5%). The lower bound was 10.4%; sensitivity estimates were 14.3% with standard IPW, 17.2% with truncated IPW, and 17.3% with outcome-model standardization. Antivenom was administered in 196/499 encounters and was not independently associated with delayed elevation (adjusted odds ratio, 1.35; 95% confidence interval, 0.68–2.68). Delayed elevation was associated with local progression (40/45 vs. 27/212; *p* < 0.001), whereas major systemic outcomes were not more frequent (2/45 vs. 15/212; *p* = 0.745). Among 39 encounters measured on hospital days 1–5, the median CK level peaked on day 3 and remained above 1000 U/L on day 5. **Conclusions:** An initially normal CK level did not exclude later elevation among retested encounters. The population incidence remained uncertain because follow-up was clinically patterned. Delayed elevation was primarily linked to local progression rather than major systemic outcomes. Selective repeat CK testing may complement serial clinical assessments when envenomation remains active.

## 1. Introduction

Snakebites are toxicologic emergencies with manifestations that may evolve after presentation. Local pain and swelling are common, whereas systemic envenomation can cause coagulopathy, acute kidney injury, shock, neurological abnormalities, and death [1,2]. Because early findings may underestimate subsequent toxicity, serial clinical assessments remain central to the management of these patients [2,3].

National emergency department studies have documented substantial hospital utilization and marked seasonal and geographic clustering of snakebites in South Korea [4,5]. Previous Korean clinical studies have characterized coagulopathy, acute kidney injury, and the broader clinical spectrum of envenomation [6,7,8]. In South Korea, medically important snakebites are predominantly attributed to Asian pit vipers of the genus *Gloydius* (family Viperidae, subfamily Crotalinae), particularly *G. brevicaudus*, *G. ussuriensis*, and *G. intermedius* [8,9]. These species may coexist within mountainous and rural catchment regions, and recent molecular studies have indicated species-related differences in their venom composition [9]. However, individual species are rarely identified during routine emergency care.

Venom-induced myotoxicity may increase serum creatine kinase (CK) activity and, in severe cases, lead to rhabdomyolysis [10,11,12]. Serum CK activity is widely used as a marker of skeletal muscle injury; however, it does not increase immediately after injury and may remain elevated for several days [10,13]. Prior snakebite studies have largely focused on established rhabdomyolysis, acute kidney injury, or severe envenomation rather than on patients with reassuring initial serum CK values [7,10,11,12]. Previous serial testing studies have also shown that clinically relevant laboratory abnormalities may emerge after an initially reassuring snakebite assessment [14]. Consequently, the frequency, clinical context, and temporal course of delayed serum CK elevation remain insufficiently characterized.

This study aimed to estimate the delayed elevation of CK after an initially normal measurement and to describe the hospital-day trajectory. We also examined the influence of incomplete follow-up, antivenom exposure and timing, and the relationship between delayed CK elevation and local progression, operations, intensive care, renal failure, dialysis, and death.

## 2. Materials and Methods

### 2.1. Study Design and Setting

This single-center retrospective observational study was conducted at a tertiary university-affiliated hospital in South Korea that operates an emergency medical and trauma center. The hospital serves a broad, mountainous, and rural catchment area. The study period was from January 2011 to June 2025.

### 2.2. Study Population

Eligible encounters had a documented history consistent with snakebite and a final diagnosis assigned by the treating emergency physician. Encounters recorded only as rule-out snakebite were excluded. Of 542 presentations, 32 were excluded because the secondary research use of the clinical data was not authorized under the institutional policy and 11 because of data-collection errors, leaving 499 eligible snakebite encounters involving 471 individuals. The encounter was the unit of analysis; some individuals contributed more than one encounter during the study period. Encounters ending in discharge against medical advice or transfer were excluded from disposition and inpatient course comparisons.

### 2.3. Initial Management and Antivenom Exposure

Patients with suspected envenomation were initially observed in the emergency department and reassessed by a board-certified emergency physician. Decisions regarding antivenom administration, admission, surgery, and intensive care were based on evolving local and systemic manifestations. The antivenom used throughout the study was Kovax freeze-dried *Agkistrodon halys* antivenom injection (6000 IU/vial; Korea Vaccine, Ansan, Republic of Korea). Institutional practice was to administer one vial per episode of administration.

Antivenom exposure was defined as any recorded administration versus none. Because each administration consisted of one vial, the recorded administration count also represented the cumulative vial count, and the cumulative dose was calculated as the administration count multiplied by 6000 IU. The administration count and cumulative dose were summarized descriptively; they were not treated as causal dose–response predictors because repeat dosing was determined by clinical severity and treatment response. When available, the interval from the bite event to the first antivenom administration was determined. A 6-h threshold, corresponding to the cohort median, was used for exploratory timing analyses.

### 2.4. Data Collection and CK Measurement Timing

Electronic medical records were reviewed for demographic characteristics, initial vital signs, laboratory findings, emergency department disposition, antivenom treatment, operations, complications, intensive care, dialysis, in-hospital death, and length of hospital stay. All CK measurements were serum CK activities reported in U/L; plasma and urine CK measurements were not analyzed. Final clinical outcomes and operation types were reconciled in a final adjudicated clinical outcome dataset derived from a detailed medical record review. Operations were categorized as fasciotomy, wound debridement, drainage, or other surgery.

The three CK fields in the primary dataset represented sequential measurements rather than tests obtained consistently on fixed hospital days. Therefore, they were termed the initial CK, first follow-up CK, and second follow-up CK. Delayed elevation was determined from either follow-up measurement. For trajectory analyses, dated CK measurements were reviewed separately and assigned to the actual hospital days.

Zero entries in the extracted laboratory fields were placeholders for unavailable results rather than measured zero concentrations or values below the assay detection limit, and were treated as missing. Snake species were not systematically confirmed by expert examination, photography, captured specimens, or molecular testing. Therefore, species were not retrospectively assigned based on geographical location or clinical manifestations.

### 2.5. Clinical Outcome Definitions

Among the admitted encounters, the clinical course was classified as simple or complex. A complex course was defined as at least one of the following: prolonged progressive wound swelling, a non-rhabdomyolysis complication, an operation, intensive care unit (ICU) admission, or death. Rhabdomyolysis was excluded from the composite to avoid circularity in CK-centered analyses. Because viperid-associated swelling may intensify over the first 2–3 days and persist thereafter [15], prolonged swelling was operationally defined as continued progression beyond 72 h.

To address the heterogeneity of the composite, encounters without a predefined local or systemic outcome were retained as the simple-course group, whereas complex-course encounters were separated into two mutually exclusive phenotypes. Local progression only comprised prolonged swelling, compartment syndrome, or an operation without a predefined systemic outcome. Systemic outcomes with or without local progression comprised ICU admission, in-hospital death, dialysis or renal replacement therapy, acute renal failure, coagulopathy, pleural effusion, hemoperitoneum, or anaphylaxis.

For direct comparisons according to delayed CK elevation, local progression was defined as prolonged swelling, compartment syndrome, or any operation. A major systemic outcome was defined as ICU admission, acute renal failure, dialysis or renal replacement therapy, or in-hospital death. A broader systemic composite additionally included coagulopathy, pleural effusion, hemoperitoneum, and anaphylaxis.

### 2.6. CK-Related Analytic Cohorts and Outcomes

An initially normal serum CK value was defined as a serum CK value <1000 U/L at the initial measurement. Delayed serum CK elevation was defined as a serum CK level ≥1000 U/L at either the first or second follow-up measurement. The 1000 U/L threshold was selected as a pragmatic analytic marker, consistent with prior snakebite myotoxicity studies and commonly applied rhabdomyolysis criteria [10,11,16]. It was not regarded as a species-specific diagnostic cutoff or a stand-alone indication for admission, antivenom therapy, surgery, or dialysis.

The primary outcome was the observed proportion of delayed CK elevation among encounters with at least one follow-up CK measurement. Secondary and exploratory outcomes included sensitivity estimates for incomplete follow-up, associations with antivenom exposure and timing, revised local and systemic phenotypes, individual clinical outcomes, and the temporal CK course during hospitalization.

Potential trajectory records were validated against dated laboratory results and actual hospital days. Records were excluded when the qualifying CK elevation occurred only after hospital day 3 or when a dated hospital-day trajectory could not be verified. Patient-level trajectory parameters included the hospital day of peak CK, the first observed decline after the peak, and the first observed post-peak CK value below 1000 U/L. The main trajectory figure was based on encounters with complete serum CK measurements on hospital days 1–5.

### 2.7. Incomplete Follow-Up CK Measurements

The complete-case analysis included encounters with initial serum CK <1000 U/L and at least one follow-up measurement. A conservative lower-bound proportion used all encounters with an initially normal serum CK value as the denominator and assigned no delayed elevation event to encounters without follow-up testing.

Because repeat testing was clinically determined, the probability of follow-up CK testing was estimated using a multivariable logistic model that included age, sex, log_2_-transformed initial serum CK, initial white blood cell count, antivenom administration, and recorded hospital admission indicator. A Hájek-normalized inverse-probability-weighted (IPW) estimate was calculated. To assess sensitivity to influential weights, a secondary estimate truncated the weights at the 99th percentile. Separately, an outcome model fitted among encounters with observed follow-up results was standardized to the full cohort with initially normal serum CK values.

The complete-case confidence interval (CI) used the Wilson method. CIs for weighted and outcome model estimates were obtained from 2000 valid nonparametric bootstrap samples, resampled by individual patient identifier to account for repeated encounters. These analyses were considered sensitivity analyses rather than definitive estimates of population incidence.

### 2.8. Statistical Analysis

Continuous variables are presented as medians with interquartile ranges (IQRs), and categorical variables as numbers with percentages. Two-group comparisons were performed using the Mann–Whitney *U* test for continuous variables and the chi-square test or Fisher’s exact test for categorical variables, as appropriate. CK variables across the three mutually exclusive phenotypes were compared using the Kruskal–Wallis test. The component variables used to define the phenotypes were reported descriptively, without circular significance testing.

Multivariable logistic regression was used to examine the association between antivenom administration and delayed CK elevation. The adjusted model included age, sex, log_2_-transformed initial CK, initial white blood cell count, and antivenom administration; standard errors were clustered by individual patient identifier. A separate exploratory model compared no antivenom, antivenom within 6 h, and antivenom after 6 h. Clinical outcome comparisons were exploratory, and no formal adjustments for multiple comparisons were applied.

All tests were two-sided, and statistical significance was set at *p* < 0.05. Original descriptive analyses were conducted using IBM SPSS Statistics for Windows, version 27.0 (IBM Corp., Armonk, NY, USA). Revised weighting, bootstrap, regression, and quality control analyses were performed in Python, version 3.13.5 (Python Software Foundation, Wilmington, DE, USA) using reproducible analysis code.

### 2.9. Ethics Statement

The study was conducted in accordance with the Declaration of Helsinki and was approved by the Institutional Review Board of Wonju Severance Christian Hospital, Yonsei University Wonju College of Medicine (IRB No. CR325016; 29 April 2025). The requirement for study-specific informed consent was waived because of the retrospective design and the use of de-identified medical record data. The waiver was applied to records eligible for secondary research use under institutional policy; records without such authorization were excluded.

### 2.10. Use of Generative Artificial Intelligence

During the preparation of this manuscript, the authors used ChatGPT (GPT-5.5 Pro and 5.6 Pro; OpenAI, San Francisco, CA, USA; accessed 21 July 2026) as a generative artificial intelligence tool to assist with English-language editing. However, the manuscript has been thoroughly reviewed and edited by a professional English language editor. The tool was not used to alter the source data or make unsupervised clinical or statistical decisions.

## 3. Results

### 3.1. Study Population and Analytic Cohorts

**Figure 1:**
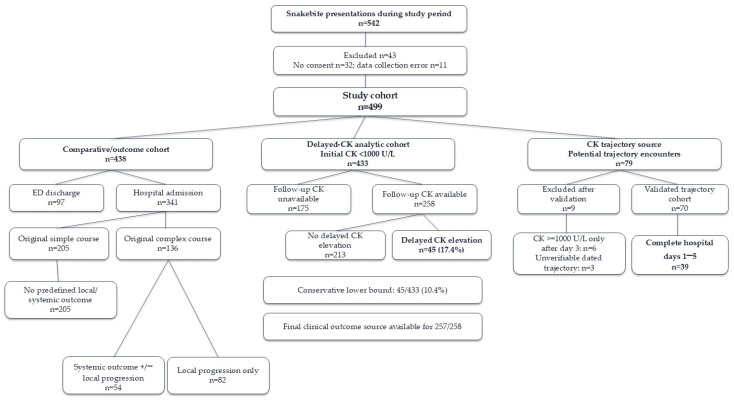
Study flow and analytic cohorts. The 499 eligible encounters involved 471 individuals. CK, serum creatine kinase; ED, emergency department.

During the study period, 542 snakebite presentations were identified. After excluding 32 encounters for which secondary research use of the clinical data was not authorized and 11 with data-collection errors, 499 eligible snakebite encounters involving 471 individuals were included in the study. Forty encounters ending in discharge against medical advice and 21 ending in transfer were excluded from the comparative analyses, leaving 438 encounters: 97 discharged from the emergency department and 341 admitted to the hospital (Figure 1).

Among the 499 encounters, 433 had initial CK levels <1000 U/L. Of these, at least one follow-up CK measurement was available for 258, whereas 175 had no follow-up measurements. The trajectory review identified 79 potential encounters, 70 validated trajectories, and 39 encounters with complete CK measurements on hospital days 1–5.

### 3.2. Baseline Characteristics According to Emergency Department Disposition

Compared with encounters discharged from the emergency department, admitted encounters had a higher heart rate (83 [72–96] vs. 78 [69–89] beats/min, *p* = 0.018), higher initial white blood cell count (7.95 [6.26–11.26] vs. 6.59 [5.72–8.31] × 10^3^/µL, *p* < 0.001), lower initial platelet count (228 [185–274] vs. 247 [200–299] × 10^3^/µL, *p* = 0.024), and higher initial CK level (162 [111–279] vs. 124 [93–182] U/L, *p* < 0.001). The remaining baseline characteristics did not differ significantly (Table 1).

### 3.3. Delayed CK Elevation and Incomplete Follow-Up

Among 433 encounters with initial CK <1000 U/L, 258 underwent repeat testing, and 45 developed delayed CK elevation. The observed complete-case proportion was 17.4% (95% CI, 13.3–22.5%), whereas the conservative lower bound was 10.4% (45/433). The standard Hájek IPW estimate was 14.3% (95% bootstrap CI, 10.6–19.8%), the 99th-percentile truncated IPW estimate was 17.2% (12.0–21.8%), and the outcome-model standardized estimate was 17.3% (12.7–22.4%) (Table 2).

Follow-up testing was strongly associated with antivenom administration (odds ratio [OR], 3.46; 95% CI, 2.10–5.71; *p* < 0.001) and admission status (OR, 58.89; 95% CI, 13.72–252.79; *p* < 0.001) (Supplementary Table S1). The maximum inverse observation weight was 44.96. The difference between the standard and truncated IPW estimates indicates sensitivity to this extreme weight; nevertheless, all sensitivity estimates remained above the conservative lower bound.

### 3.4. Antivenom Exposure and Delayed CK Elevation

Antivenom was administered in 196 of 499 encounters (39.3%). Among the recipients, the median administration count was 1 (IQR, 1–2), corresponding to a median cumulative dose of 6000 IU (IQR, 6000–12,000 IU). Delayed CK elevation occurred in 18 of 117 encounters without antivenom (15.4%) and 27 of 141 with antivenom (19.1%; *p* = 0.510). Antivenom administration was not independently associated with delayed elevation after adjustment (OR, 1.35; 95% CI, 0.68–2.68; *p* = 0.391) (Table 3).

Bite-to-first-antivenom timing was available in 193 of 196 treated encounters, with a median of 6 h (IQR, 3–12 h). Delayed elevation occurred in 9/60 encounters treated within 6 h (15.0%) and 18/78 treated after 6 h (23.1%), compared with 18/117 without antivenom (15.4%; three-group *p* = 0.319). The adjusted timing estimates and antivenom-stratified trajectory summaries are provided in Supplementary Tables S2 and S4, respectively.

### 3.5. Revised Clinical Outcome Phenotypes

The final clinical outcome dataset reproduced the original simple- and complex-course classifications as 205 and 136 encounters, respectively. After separation of the heterogeneous complex group, 82 encounters had local progression only and 54 had a systemic outcome, with or without local progression. The components were not mutually exclusive. Prolonged swelling occurred in 75 encounters, compartment syndrome in 22, and an operation in 23 (11 fasciotomies, seven wound debridements, three drainage procedures, and two other operations). Systemic components included ICU admission in 11, in-hospital death in 11, dialysis or renal replacement therapy in three, acute renal failure in 16, coagulopathy in 40, pleural effusion in two, hemoperitoneum in one, and anaphylaxis in five. Sixty-six encounters had at least one non-rhabdomyolysis complication (Table 4A).

CK levels differed across the revised phenotypes (Table 4B). The median first follow-up CK was 127 U/L (IQR, 82.5–243.5; n = 163) in encounters with no predefined local or systemic outcomes, 1072 U/L (263.5–2886; n = 71) in those with local progression only, and 199 U/L (111–1151.2; n = 46) in those with a systemic outcome with or without local progression (*p* < 0.001). The second follow-up CK and the change from the initial to the first follow-up CK showed the same pattern. The median hospital stays were 3, 6, and 6 days, respectively (*p* < 0.001).

### 3.6. Clinical Outcomes According to Delayed CK Elevation

Final clinical outcome data were available for 257 of the 258 encounters in the primary delayed-CK cohort; unmatched encounters had no delayed elevation. Local progression was documented in 40/45 (88.9%) encounters with delayed CK elevation and 27/212 (12.7%; *p* < 0.001) without delayed elevation. Prolonged swelling accounted for most of this difference (40/45 [88.9%] vs. 6/212 [2.8%]; *p* < 0.001) (Table 5).

Major systemic outcomes were not more frequent with delayed elevation (2/45 [4.4%] vs. 15/212 [7.1%]; *p* = 0.745). Fasciotomy occurred in 1/45 versus 5/212, ICU admission in 2/45 versus 5/212, in-hospital death in 1/45 versus 6/212, acute renal failure in 1/45 versus 10/212, and dialysis in 1/45 versus 1/212. Among the admitted encounters, the median hospital stay was 5 days (IQR, 4–6.2; n = 44) with delayed elevation and 4 days (3–6; n = 207) without delayed elevation (*p* = 0.001).

### 3.7. Hospital-Day CK Trajectory

The validated trajectory cohort comprised 70 encounters with CK ≥1000 U/L within actual hospital days 1–3. Peak CK occurred on a median of hospital day 3 (IQR, 1–3; n = 70). The first observed decline occurred on day 4 (IQR, 3–5; n = 66), and the first observed post-peak CK value below 1000 U/L occurred on day 6 (IQR, 5–8; n = 52) (Supplementary Table S3).

**Figure 2:**
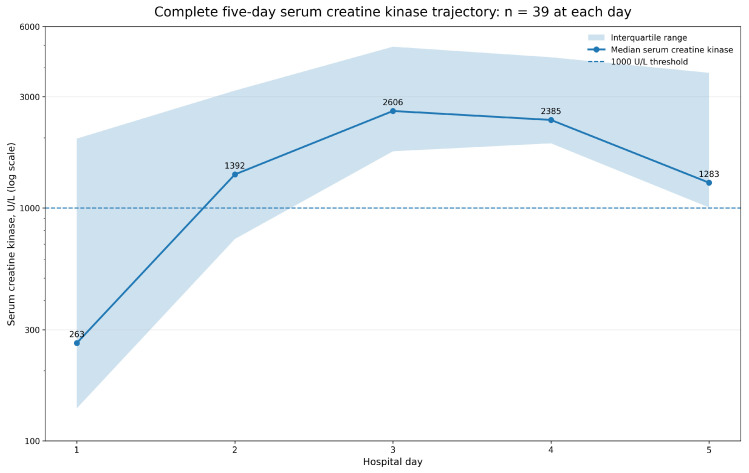
Serum creatine kinase trajectory in the complete five-day cohort. The medians and interquartile ranges are shown for the same 39 encounters with CK measurements on each of hospital days 1–5. These encounters were drawn from the validated trajectory cohort, defined by serum CK ≥1000 U/L within hospital days 1–3. The dashed line indicates the operational analytic threshold of 1000 U/L. CK, serum creatine kinase.

Figure 2 shows the same 39 encounters with measurements taken every hospital day from days 1 to 5. Median CK values were 263 U/L (IQR, 138–1983.5) on day 1, 1392 U/L (735.5–3188) on day 2, 2606 U/L (1750–4919.5) on day 3, 2385 U/L (1893.5–4431.5) on day 4, and 1283 U/L (1004.5–3801) on day 5. Thus, the median reached its maximum on day 3 and remained above the operational threshold on day 5.

## 4. Discussion

This study shows that a normal CK value at the initial assessment does not invariably exclude later biochemical evidence of muscle injury after a snakebite. Delayed elevation occurred in approximately one out of six encounters selected for repeat testing. This phenomenon remained evident across several approaches to incomplete follow-up, although the range of weighted estimates and extreme observation weights precluded a single definitive population rate. Clinically, delayed elevation was more strongly associated with ongoing local progression than with intensive care, renal failure, dialysis, surgery, or death.

This finding is biologically plausible because CK is released after the disruption of skeletal-muscle cell membranes and rises later than the initiating tissue injury [10,13]. Therefore, venom-associated muscle injury may evolve while the initial circulating CK concentration remains below the conventional threshold. This differs from studies that begin with established rhabdomyolysis or organ injury; the present analysis focuses on the earlier diagnostic window in which the first CK result may appear reassuring [7,10,11,12].

The hospital-day trajectory adds temporal context. In the fixed cohort of 39 encounters, the median CK level rose through to hospital day 3 and remained >1000 U/L on day 5. In the broader validated cohort, the first observed decline occurred on a median of day 4, and the first observed post-peak value <1000 U/L occurred on day 6. These findings extend prior serial testing research by focusing specifically on delayed serum CK elevation after an initially normal result [14]. These observations should not be interpreted as a universal timetable because prolonged testing was concentrated among admitted patients with continued clinical concern. However, they do indicate that CK can lag behind the initial examination and that a single early value cannot define the subsequent laboratory course.

Distinguishing between local and systemic outcomes also clarifies what CK represents and what it does not. Local swelling is a venom-mediated inflammatory manifestation and should not be considered interchangeable with systemic envenomation severity [17]. Follow-up CK values were highest in the local-progression-only phenotype, and delayed elevation predominantly occurred in encounters with prolonged swelling. In contrast, severe systemic toxicity was heterogeneous and could occur without marked CK elevation. The cohort included ICU admissions, in-hospital deaths, acute renal failure, and dialysis; however, the major systemic composite was not more frequent among encounters with delayed CK elevation. Therefore, CK should be viewed as a marker of muscle injury that complements, but does not replace, the assessment of bleeding, coagulopathy, renal function, hemodynamics, neurological status, and local tissue viability.

This distinction is relevant for clinical monitoring. The results do not support routine daily CK measurements or prolonged admission for every snakebite. Repeat testing is more defensible when swelling continues to progress, pain or weakness worsens, urine or renal findings become abnormal, the patient presents early after the bite, or other evidence of active envenomation persists. The 1000 U/L threshold was useful for reproducible analysis but is not an independent trigger for antivenom, fasciotomy, dialysis, or discharge decisions.

Antivenom exposure was not independently associated with delayed CK elevation, and no statistically significant difference was detected among untreated, ≤6-h, and >6-h groups. This result cannot establish therapeutic equivalence. Antivenom was administered according to evolving severity, and patients with more pronounced envenomation were also more likely to receive repeated doses and follow-up CK testing. Such confounding by indication, together with species heterogeneity and modest subgroup sizes, limits the causal interpretation. The administration count and cumulative dose describe treatment intensity but should not be interpreted as a randomized dose–response comparison.

The incomplete follow-up remains important. Repeated CK measurements were strongly associated with admission and antivenom treatment, confirming a clinical pattern in the missing data. Therefore, the complete-case proportion applies to the retested subgroup, whereas the lower bound and model-based estimates address different assumptions. The shift from 14.3% with standard IPW to 17.2% after truncating an extreme weight illustrates the model’s sensitivity. Taken together, these analyses support the presence of delayed CK elevation but do not identify its exact incidence among all encounters with an initially normal value.

The toxicologic context also limits interpretation. Medically important snakebites in South Korea are predominantly attributed to *Gloydius* pit vipers, including *G. brevicaudus*, *G. ussuriensis*, and *G. intermedius* [8,9]. These species can differ in their venom composition and may coexist within the catchment region. Because individual species were not confirmed, the results should be interpreted as syndrome-based findings from a Korean *Gloydius*-dominant setting, rather than as species-specific estimates.

### Strengths and Limitations

The strengths of this study include a large, long-term real-world cohort; linkage of sequential CK measurements to adjudicated clinical outcomes; direct characterization of operations, intensive care, in-hospital death, renal failure, dialysis, and complications; explicit analyses of antivenom exposure and cumulative treatment; a fixed denominator trajectory; and complementary approaches to incomplete follow-up data. Explicit definitions, clustered resampling, and transparent denominator reporting improve reproducibility and interpretation.

However, this study has some limitations. The retrospective, single-center design produced non-standardized sampling and substantial selection for repeat testing. Weighting and outcome-model standardization address measured predictors but cannot remove unmeasured confounding or model misspecification, and the standard IPW result was sensitive to an extreme weight. Antivenom was not randomly allocated, and cumulative treatment reflected evolving severity. The 499 encounters were contributed by 471 individuals, and regression and bootstrap procedures accounted for within-person clustering. One encounter in the primary CK cohort could not be linked to the final clinical outcome dataset. Major systemic events were uncommon, limiting precision, and individual clinical outcome comparisons were exploratory in nature. Furthermore, species were not confirmed, the CK threshold was operational, and the complete 5-day trajectory represented a selected hospitalized subgroup.

Prospective multicenter studies should use prespecified CK sampling intervals, standardized measures of local and systemic severity, verified species identification where feasible, and protocolized recording of antivenom timing and dosage. Such studies could determine which combinations of clinical findings and laboratory trends most efficiently identify patients who would benefit from repeat testing or prolonged observation.

## 5. Conclusions

Among encounters selected for repeat testing after an initially normal CK value, delayed elevation was not uncommon. Sensitivity analyses addressing incomplete follow-up supported the persistence of this phenomenon but did not establish a single population incidence rate. Delayed CK elevation was predominantly associated with ongoing local progression rather than major systemic outcomes. The median CK level peaked on hospital day 3 in the complete 5-day cohort. Selective follow-up CK measurements may complement serial clinical assessments when envenomation remains active; however, CK should not be used alone to define severity or treatment need.

## Figures and Tables

**Table 1:** Baseline characteristics according to emergency department disposition.

Variable	ED Discharge (n = 97)	Admission (n = 341)	*p*-Value
Age, years	58 (49–67)	59 (48–69)	0.807
Male sex, n/N (%)	63/97 (64.9%)	217/341 (63.6%)	0.906
SBP, mmHg	150 (131–162)	144 (127–159)	0.094
HR, beats/min	78 (69–89)	83 (72–96)	0.018
RR, breaths/min	18 (18–20)	18 (18–20)	0.645
Body temperature, °C	36.7 (36.4–36.9)	36.7 (36.4–37.0)	0.291
Initial WBC, ×10^3^/µL	6.59 (5.72–8.31)	7.95 (6.26–11.26)	<0.001
Initial hemoglobin, g/dL	14.2 (13.4–15.2)	14.4 (13.4–15.4)	0.375
Initial platelet count, ×10^3^/µL	247 (200–299)	228 (185–274)	0.024
Initial creatinine, mg/dL	0.78 (0.64–0.99)	0.80 (0.66–0.96)	0.690
Initial LDH, U/L	230 (206–266)	243 (214–288)	0.056
Initial CK, U/L	124 (93–182)	162 (111–279)	<0.001

Values are median (interquartile range) unless otherwise indicated. Zero entries in the extracted laboratory fields represented unavailable results rather than measured concentrations and were treated as missing. CK, serum creatine kinase; ED, emergency department; HR, heart rate; LDH, lactate dehydrogenase; RR, respiratory rate; SBP, systolic blood pressure; WBC, white blood cell count.

**Table 2:** Delayed CK elevation and sensitivity analyses for incomplete follow-up.

Analysis	Numerator/Denominator or Target Population	Estimate	95% CI
Observed complete-case proportion	45/258	17.4%	13.3–22.5%
Conservative lower-bound proportion	45/433	10.4%	Not applicable
IPW-adjusted estimate (Hájek)	Target n = 433	14.3%	10.6–19.8%
99th-percentile truncated IPW estimate	Target n = 433	17.2%	12.0–21.8%
Outcome-model standardized estimate	Target n = 433	17.3%	12.7–22.4%

The complete-case proportion applies to the retested subgroup. The lower bound assigned no delayed elevation to all encounters without follow-up CK. No CI was calculated for this deterministic lower bound. Weighted and outcome model estimates used 2000 valid bootstrap samples resampled using individual patient identifiers. Standard IPW was sensitive to an extreme weight (maximum, 44.96); the truncated estimate capped weights at the 99th percentile. CI, confidence interval; IPW, inverse-probability weighting.

**Table 3:** Antivenom exposure and delayed CK elevation.

**A. Antivenom Administration in the Study and Primary-Analysis Cohorts**			
**Measure**	**No Antivenom**	**Any Antivenom**	***p*** **-Value/Estimate**
Entire study cohort	303/499 (60.7%)	196/499 (39.3%)	
Primary delayed-CK cohort	117	141	
Delayed CK elevation	18/117 (15.4%)	27/141 (19.1%)	*p* = 0.510
Administration count, median (IQR)		1 (1–2)	1 administration = 1 vial
Cumulative dose, IU, median (IQR)		6000 (6000–12,000)	6000 IU/vial
Bite-to-first-antivenom time, h, median (IQR)		6 (3–12)	Available in 193/196
**B. Multivariable Logistic Regression for Delayed CK Elevation**			
**Covariate**	**Adjusted OR (95% CI)**		***p*** **-Value**
Age, per year	0.99 (0.98–1.01)		0.538
Male sex	2.24 (0.96–5.18)		0.061
Initial CK, per log2 increase	1.08 (0.66–1.75)		0.756
Initial WBC, per 1 × 10^3^/µL	0.96 (0.88–1.03)		0.260
Any antivenom administration	1.35 (0.68–2.68)		0.391

Values are n/N (%) or median (interquartile range), unless otherwise indicated. Each antivenom administration consisted of one 6000-IU vial. Administration counts and cumulative doses are descriptive treatment measures. The adjusted model used standard errors clustered by individual patient identifiers. CI, confidence interval; CK, serum creatine kinase; IQR, interquartile range; IU, international unit; OR, odds ratio; WBC, white blood cell count.

**Table 4:** Revised clinical outcome phenotypes among admitted encounters.

**A. Outcome Classification and Component Counts**				
**Characteristic**	**No Predefined Local/Systemic Outcome (n = 205)**	**Local Progression Only (n = 82)**	**Systemic Outcome ± Local Progression (n = 54)**	
Original simple course	205/205 (100.0%)	0/82 (0.0%)	0/54 (0.0%)	
Original complex course	0/205 (0.0%)	82/82 (100.0%)	54/54 (100.0%)	
Prolonged wound swelling	0/205 (0.0%)	60/82 (73.2%)	15/54 (27.8%)	
Compartment syndrome	0/205 (0.0%)	14/82 (17.1%)	8/54 (14.8%)	
Any operation	0/205 (0.0%)	17/82 (20.7%)	6/54 (11.1%)	
Fasciotomy	0/205 (0.0%)	8/82 (9.8%)	3/54 (5.6%)	
Wound debridement	0/205 (0.0%)	6/82 (7.3%)	1/54 (1.9%)	
Drainage	0/205 (0.0%)	3/82 (3.7%)	0/54 (0.0%)	
Other operation	0/205 (0.0%)	0/82 (0.0%)	2/54 (3.7%)	
Any non-rhabdomyolysis complication	0/205 (0.0%)	14/82 (17.1%)	52/54 (96.3%)	
ICU admission	0/205 (0.0%)	0/82 (0.0%)	11/54 (20.4%)	
In-hospital death	0/205 (0.0%)	0/82 (0.0%)	11/54 (20.4%)	
Dialysis/renal replacement therapy	0/205 (0.0%)	0/82 (0.0%)	3/54 (5.6%)	
Acute renal failure	0/205 (0.0%)	0/82 (0.0%)	16/54 (29.6%)	
Coagulopathy	0/205 (0.0%)	0/82 (0.0%)	40/54 (74.1%)	
Pleural effusion	0/205 (0.0%)	0/82 (0.0%)	2/54 (3.7%)	
Hemoperitoneum	0/205 (0.0%)	0/82 (0.0%)	1/54 (1.9%)	
Anaphylaxis	0/205 (0.0%)	0/82 (0.0%)	5/54 (9.3%)	
**B. CK-Related Variables According to Mutually Exclusive Phenotypes**				
**Variable**	**No Predefined Local/Systemic Outcome** **(n = 205)**	**Local Progression Only** **(n = 82)**	**Systemic Outcome ± Local Progression (n = 54)**	***p*** **-Value**
Initial CK, U/L	144 (106–221.2) [n = 196]	245.5 (126.5–785.2) [n = 80]	200 (120–375) [n = 53]	<0.001
First follow-up CK, U/L	127 (82.5–243.5) [n = 163]	1072 (263.5–2886) [n = 71]	199 (111–1151.2) [n = 46]	<0.001
Second follow-up CK, U/L	157 (89–427) [n = 117]	1805 (487–3972.2) [n = 62]	250 (107.5–1256) [n = 39]	<0.001
Change from initial to first follow-up CK, U/L	−29 (−54.5–21) [n = 163]	141 (−56.5–1296.5) [n = 71]	−16 (−57–87) [n = 45]	<0.001
Total hospital stay, days	3 (2–5) [n = 204]	6 (4.2–8) [n = 82]	6 (4–10.8) [n = 54]	<0.001

Panel A reports the variables used to define the phenotypes and therefore does not include *p*-values. Components are not mutually exclusive. Rhabdomyolysis was excluded from the phenotype definitions. Panel B values are median (interquartile range); bracketed n values are available denominators. CK, serum creatine kinase; ICU, intensive care unit.

**Table 5:** Clinical outcomes according to delayed CK elevation in the primary-analysis cohort.

Outcome	No Delayed CK Elevation (n = 212)	Delayed CK Elevation (n = 45)	*p*-Value
Local progression	27/212 (12.7%)	40/45 (88.9%)	<0.001
Prolonged wound swelling	6/212 (2.8%)	40/45 (88.9%)	<0.001
Compartment syndrome	18/212 (8.5%)	0/45 (0.0%)	0.049
Any operation	10/212 (4.7%)	1/45 (2.2%)	0.695
Fasciotomy	5/212 (2.4%)	1/45 (2.2%)	1.000
Wound debridement	4/212 (1.9%)	0/45 (0.0%)	1.000
Drainage	1/212 (0.5%)	0/45 (0.0%)	1.000
Other operation	0/212 (0.0%)	0/45 (0.0%)	—
ICU admission	5/212 (2.4%)	2/45 (4.4%)	0.354
In-hospital death	6/212 (2.8%)	1/45 (2.2%)	1.000
Acute renal failure	10/212 (4.7%)	1/45 (2.2%)	0.695
Dialysis/renal replacement therapy	1/212 (0.5%)	1/45 (2.2%)	0.320
Coagulopathy	26/212 (12.3%)	3/45 (6.7%)	0.435
Pleural effusion	0/212 (0.0%)	0/45 (0.0%)	—
Hemoperitoneum	0/212 (0.0%)	0/45 (0.0%)	—
Anaphylaxis	2/212 (0.9%)	1/45 (2.2%)	0.440
Major systemic outcome	15/212 (7.1%)	2/45 (4.4%)	0.745
Broader systemic outcome composite	31/212 (14.6%)	5/45 (11.1%)	0.642
Total hospital stay among admitted encounters, days	4 (3–6) [n = 207]	5 (4–6.2) [n = 44]	0.001

Clinical outcome data were available for 257 of the 258 primary-cohort encounters; one encounter without a delayed elevation could not be linked to the final clinical outcome dataset. Major systemic outcomes included ICU admission, acute renal failure, dialysis/renal replacement therapy, and in-hospital death. The broader composite also included coagulopathy, pleural effusion, hemoperitoneum, and anaphylaxis. Individual outcome analyses were exploratory and not adjusted for multiple comparisons. No statistical tests were performed for outcomes with zero events in both groups. CK, serum creatine kinase; ICU, intensive care unit.

## Data Availability

The clinical dataset is not publicly available because of privacy and institutional review board restrictions. A de-identified analytic dataset, data dictionary, and analysis code may be made available from the corresponding author upon reasonable request, subject to institutional approval.

## Supplementary Materials

The following supporting information can be downloaded at: https://www.mdpi.com/article/10.3390/jcm15156079/s1. Table S1A, multivariable model for follow-up CK availability; Table S1B, inverse-probability weight diagnostics; Table S2A, delayed CK elevation according to bite-to-antivenom timing; Table S2B, adjusted timing model; Table S2C, antivenom administration count and cumulative dose; Table S2D, fully adjusted model for delayed CK elevation; Table S3, hospital-day CK trajectories; and Table S4, trajectory timing according to antivenom timing.

## Abbreviations

**Abbreviation**	**Definition**
CI	Confidence interval
CK	Serum creatine kinase
ED	Emergency department
HR	Heart rate
ICU	Intensive care unit
IPW	Inverse-probability weighting
IQR	Interquartile range
IU	International unit
LDH	Lactate dehydrogenase
OR	Odds ratio
RR	Respiratory rate
SBP	Systolic blood pressure
WBC	White blood cell count
