# Supplementary material for: Delayed Serum Creatine Kinase Elevation After Snakebite: Serial Changes and Clinical Correlates of Myotoxicity in a Korean Tertiary-Care Cohort
Source: J Clin Med. 2026 Aug 5;15(15):6079. doi: 10.3390/jcm15156079 (PMC13467186; doi:10.3390/jcm15156079)
Supplement: Supplementary file 1 [file jcm-15-06079-s001.zip › jcm-4446438-supplementary.pdf]

Supplementary Tables and Analyses

Supplementary Table S1A. Multivariable model for availability of follow-up serum CK testing

Predictor	OR (95% CI)	p-value
Age, per year	1.00 (0.99–1.02)	0.751
Male sex	0.67 (0.39–1.16)	0.151
Initial CK, per log_2 increase	1.12 (0.81–1.53)	0.491
Initial WBC, per 1 × 10^3/μL	1.03 (0.96–1.10)	0.393
Any antivenom administration	3.46 (2.10–5.71)	<0.001
Recorded hospital-admission indicator	58.89 (13.72–252.79)	<0.001

The model was fitted among encounters with initial serum CK <1000 U/L and used to estimate inverse observation probabilities. CI, confidence interval; CK, serum creatine kinase; OR, odds ratio; WBC, white blood cell count.

Supplementary Table S1B. Inverse-probability weight diagnostics

Weight diagnostic	Value
Observation-model sample	433
Observed follow-up records in model	258
Median inverse observation weight	1.24
95th percentile weight	1.84
99th percentile weight	1.97
Maximum weight	44.96
Valid bootstrap replications	2,000
Bootstrap attempts	2,054
Bootstrap resampling unit	Individual patient identifier

The truncated sensitivity analysis capped inverse observation weights at the 99th percentile. Bootstrap resampling was clustered by individual patient identifier.

Supplementary Table S2A. Delayed serum CK elevation according to bite-to-first-antivenom timing

Antivenom timing category	Primary-cohort, N	Delayed CK elevation	Percent
No antivenom	117	18/117	15.4%
Antivenom ≤6 h	60	9/60	15.0%
Antivenom >6 h	78	18/78	23.1%
Timing unavailable	3	0/3	0.0%
Three-group comparison			p = 0.319

The 6-h threshold corresponded to the median bite-to-first-antivenom time among recipients with timing data. CK, serum creatine kinase.

Supplementary Table S2B. Adjusted timing model for delayed serum CK elevation

Predictor	Adjusted OR (95% CI)	p-value
Antivenom ≤6 h vs. no antivenom	0.98 (0.41–2.37)	0.963
Antivenom >6 h vs. no antivenom	1.75 (0.81–3.77)	0.152
Model sample	n = 255	Three recipients with unavailable timing excluded

The model additionally included age, sex, log_2-transformed initial CK, and initial WBC. CI, confidence interval; CK, serum creatine kinase; OR, odds ratio; WBC, white blood cell count.

Supplementary Table S2C. Antivenom administration count and cumulative dose

Administration count / cumulative vials	Recipients	Cumulative dose
1	123/196 (62.8%)	6,000 IU
2	47/196 (24.0%)	12,000 IU
3	15/196 (7.7%)	18,000 IU
4	6/196 (3.1%)	24,000 IU
5	5/196 (2.6%)	30,000 IU

One administration consisted of one vial containing 6,000 IU. Administration count and cumulative dose were summarized descriptively and were not interpreted as causal dose-response measures. IU, international unit.

Supplementary Table S2D. Full adjusted model for delayed serum CK elevation

Predictor	Adjusted OR (95% CI)	p-value
Age, per year	0.99 (0.98–1.01)	0.538
Male sex	2.24 (0.96–5.18)	0.061
Initial CK, per log_2 increase	1.08 (0.66–1.75)	0.756
Initial WBC, per 1 × 10^3/μL	0.96 (0.88–1.03)	0.260
Any antivenom administration	1.35 (0.68–2.68)	0.391

Clustered standard errors accounted for repeated encounters contributed by the same individual. CI, confidence interval; CK, serum creatine kinase; OR, odds ratio; WBC, white blood cell count.

Supplementary Table S3. Hospital-day serum CK trajectories

Hospital day	Available cases, N	Median CK (IQR), U/L
Day 1	70	352.5 (180.8–2857)
Day 2	58	1497 (735–3411.5)
Day 3	61	2453 (1256–5428)
Day 4	56	2221 (1524.8–4378.5)
Day 5	51	1343 (936–3477.5)
Day 6	37	877 (404–2375)
Day 7	20	277.5 (137.2–1444.2)
Day 8	21	225 (181–632)
Day 9	7	191 (150.5–271.5)
Day 10	7	168 (115–195)
Day 11	3	158 (119–171)
Complete hospital-day 1–5 cohort	N = 39 at each day	Median CK (IQR), U/L
Day 1	39	263 (138–1983.5)
Day 2	39	1392 (735.5–3188)
Day 3	39	2606 (1750–4919.5)
Day 4	39	2385 (1893.5–4431.5)
Day 5	39	1283 (1004.5–3801)
Patient-level timing parameter	Evaluable N	Median hospital day (IQR)
Hospital day of peak CK	70	3 (1–3)
First observed decline after peak	66	4 (3–5)
First observed post-peak CK <1000 U/L	52	6 (5–8)

Available-case daily summaries use changing denominators. The complete 5-day cohort includes the same 39 encounters at each time point. CK, serum creatine kinase; IQR, interquartile range.

Supplementary Table S4. Patient-level trajectory timing according to antivenom timing

Timing parameter	No antivenom	Antivenom ≤6 h	Antivenom >6 h
Hospital day of peak CK	2 (1–3.2); n = 32	3 (1–4); n = 15	3 (2–3); n = 23
First observed decline after peak	4 (3–5); n = 31	4 (3–5); n = 14	4 (3–4); n = 21
First observed post-peak CK <1000 U/L	6 (4.8–8); n = 24	6 (3.2–7.5); n = 10	6 (6–8); n = 18
Group size	32	15	23

Values are median hospital day (interquartile range) with the evaluable denominator. Timing groups were defined using bite-to-first-antivenom time. CK, serum creatine kinase.

Supplementary Analysis Notes

The eligible snakebite encounter was the unit of analysis. Regression and bootstrap procedures accounted for repeated encounters by clustering at the individual patient-identifier level. Clinical-outcome analyses included 257 of 258 primary-cohort encounters. Individual clinical-outcome comparisons were exploratory and unadjusted for multiple comparisons. CK denotes serum creatine kinase throughout.
